# Supplementary material for: Real-time estimation of the effective reproduction number of SARS-CoV-2 in Aotearoa New Zealand
Source: PeerJ. 2022 Oct 17;10:e14119. doi: 10.7717/peerj.14119 (PMC9583856; doi:10.7717/peerj.14119)
Supplement: Supplemental Information 1 [file peerj-10-14119-s001.docx]

**Supplementary Material. Binny et al (2022).** **Real-time estimation of the effective reproduction number in Aotearoa New Zealand.**


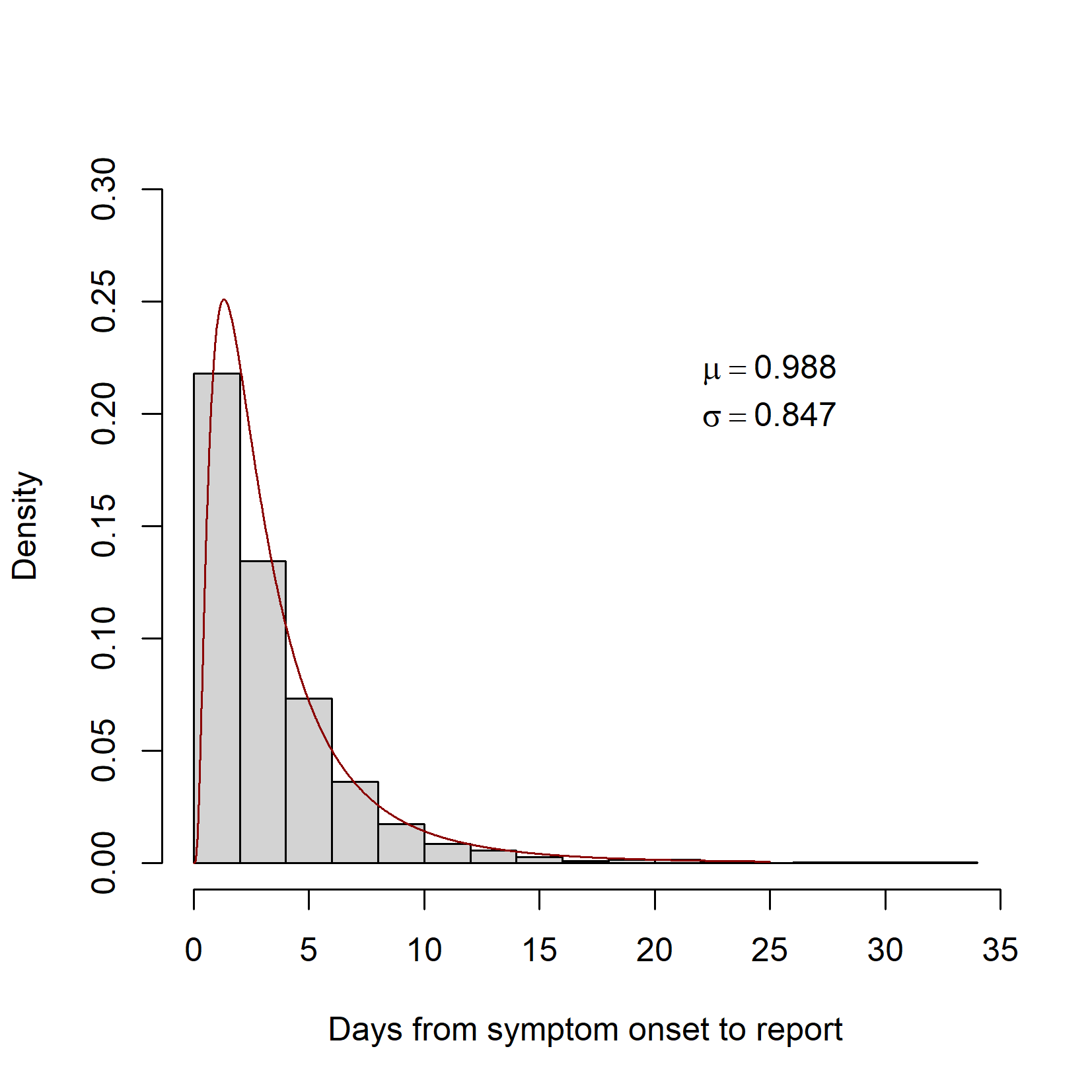


*Figure S1. Distribution of delays from the date of symptom onset to date of report. For input to the model, a report delay distribution was estimated using EpiNow2’s estimate_delay function (Abbott et al, 2020b). A log-normal distribution (red curve) was fitted to 100 subsampled bootstraps of the onset-to-report delays from 3208 cases reported between 17 August 2021 and 18 January 2022 (excluding 9 cases with delays greater than 60 days), taking (with replacement) 250 samples from these delays in each bootstrap. Using the posterior samples, the parameters of the log-normal distribution were estimated to be μ = 0.988 (SD = 0.088) and σ = 0.847 (SD = 0.063).*

*Table S2. Parameters (μ and σ) of the log-normal distribution fitted to onset-to-report delays for each month independently from 17 August 2021 – 18 January 2022.*

| **Month of report** | ***μ*** | ***σ*** |
| --- | --- | --- |
| 17/08/2021 – 16-09/2021 | 0.979 (SD 0.084) | 0.854 (SD 0.067) |
| 17/09/2021 – 16-10/2021 | 0.904 (SD 0.086) | 0.898 (SD 0.072) |
| 17/10/2021 – 16/11/2021 | 0.988 (SD 0.077) | 0.799 (SD 0.068) |
| 17/11/2021 – 16/12/2021 | 1.010 (SD 0.085) | 0.876 (SD 0.067) |
| 17/12/2021 – 18/01/2022 | 1.071 (SD 0.076) | 0.806 (SD 0.059) |

*
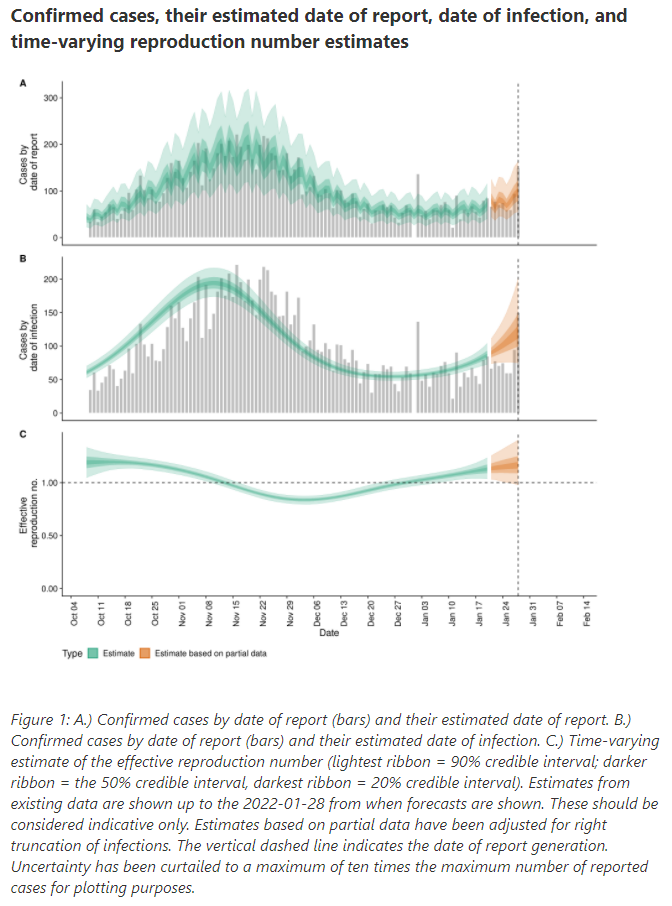

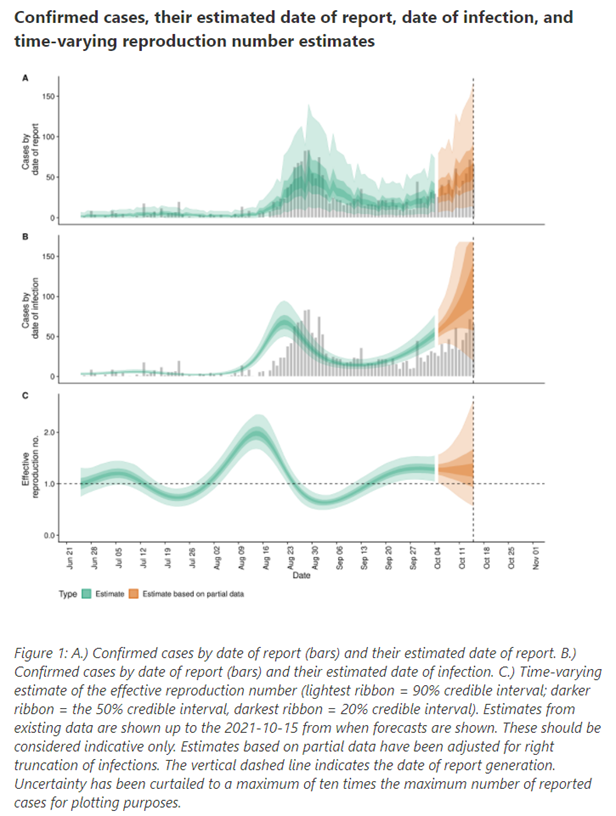
*

*Figure S2. Figure (available under Creative Commons Attribution*[*CC BY 4.0*](https://creativecommons.org/licenses/by/4.0/)*) reproduced from EpiForecasts (Abbott et al, 2020a,b, accessed from* [*https://epiforecasts.io/covid/posts/national/new-zealand/*](https://epiforecasts.io/covid/posts/national/new-zealand/) *on 19 October 2021 (left) and on 1 February 2022 (right). New cases by date of report (A), date of infection (B) and effective reproduction number (C) over 16-week periods up to 15 October 2021 (left) and up to 28 January 2022 (right).*

*
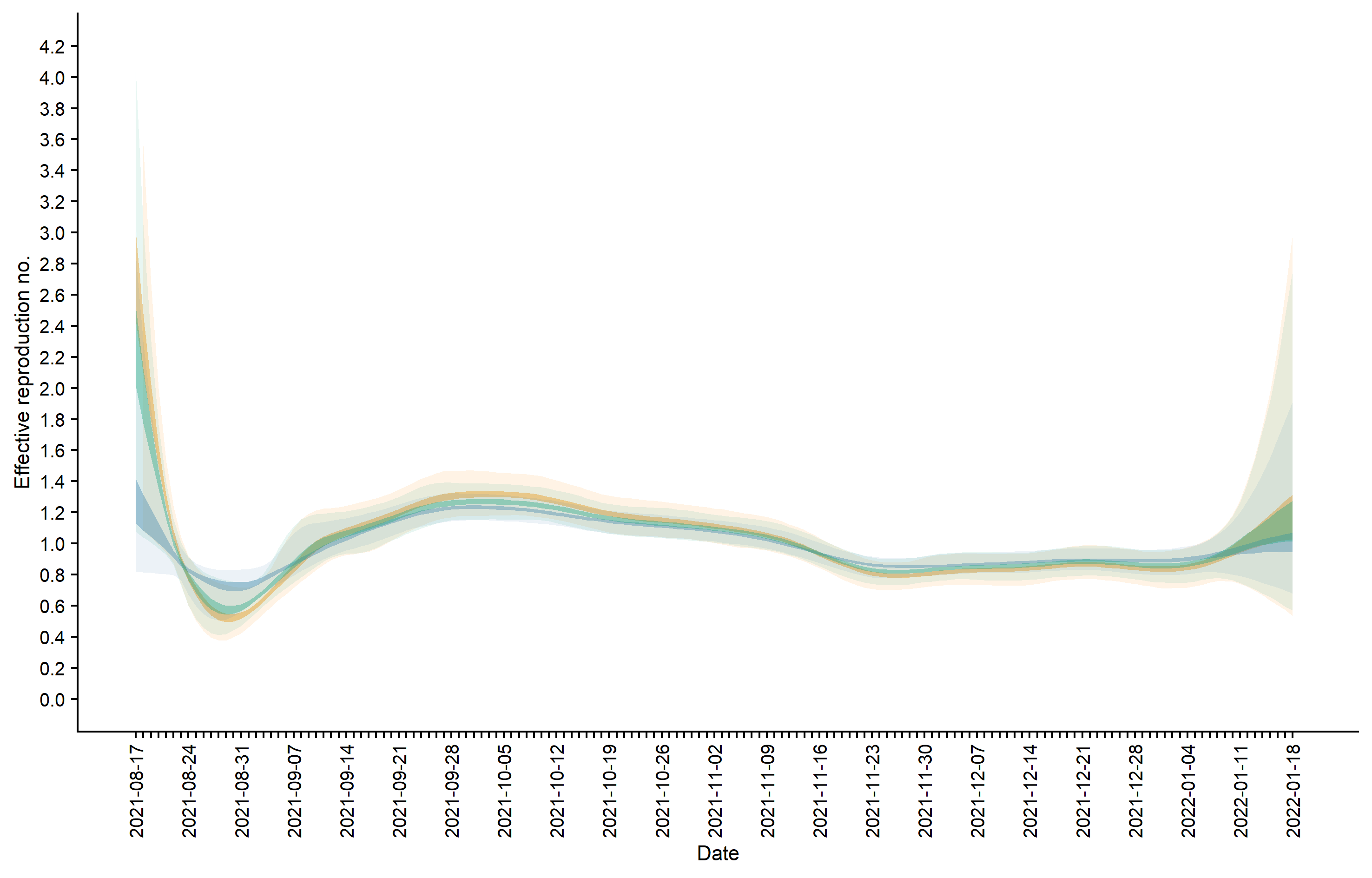
*

*Figure S3: Estimated effective reproduction number, R*_t_*, over time (light ribbon = 90% credible interval [CrI]; dark ribbon = 20% CrI) using a gamma-distributed generation time with mean of 3.6 days and SD of 3.1 days (green); a shorter mean 3.2 days and SD 2.4 days (blue); and a longer mean 4.6 days and SD 3.1 days (orange). Estimates up to 9 January 2022 are based on full data; estimates from 10 January to 18 January are based on partial data and have been adjusted for right truncation of infections.*
